# Zebrafish yolk lipid processing: a tractable tool for the study of vertebrate lipid transport and metabolism

**DOI:** 10.1242/dmm.015800

**Published:** 2014-05-08

**Authors:** Rosa L. Miyares, Vitor B. de Rezende, Steven A. Farber

**Affiliations:** 1 Department of Embryology, Carnegie Institution for Science, Baltimore, MD 21218, USA.; 2 Department of Biology, Johns Hopkins University, Baltimore, MD 21218, USA.; 3 Department of Mental Health, School of Medicine of Federal University of Minas Gerais, 30130-100 Belo Horizonte, Brazil.

**Keywords:** Dyslipidemia, Lipoprotein, Yolk syncytial layer, Yolk sac, Placenta, Lecithotrophic

## Abstract

Dyslipidemias are a major cause of morbidity and mortality in the world, particularly in developed nations. Investigating lipid and lipoprotein metabolism in experimentally tractable animal models is a crucial step towards understanding and treating human dyslipidemias. The zebrafish, a well-established embryological model, is emerging as a notable system for studies of lipid metabolism. Here, we describe the value of the lecithotrophic, or yolk-metabolizing, stages of the zebrafish as a model for studying lipid metabolism and lipoprotein transport. We demonstrate methods to assay yolk lipid metabolism in embryonic and larval zebrafish. Injection of labeled fatty acids into the zebrafish yolk promotes efficient uptake into the circulation and rapid metabolism. Using a genetic model for abetalipoproteinemia, we show that the uptake of labeled fatty acids into the circulation is dependent on lipoprotein production. Furthermore, we examine the metabolic fate of exogenously delivered fatty acids by assaying their incorporation into complex lipids. Moreover, we demonstrate that this technique is amenable to genetic and pharmacologic studies.

## INTRODUCTION

Dyslipidemias, or abnormal concentrations of serum lipids, are important risk factors for coronary artery disease, which takes the lives of more than 385,000 Americans annually (Kochanek, 2011). Approximately one-third of American adults (33.5%) have high levels of low-density lipoprotein (LDL) cholesterol (CDC, 2011). Lifestyle choices, such as diet and exercise, are contributing factors to dyslipidemias but there is also a strong genetic basis. Recently, there have been successful large-scale genome-wide association (GWA) studies in humans that have identified 95 significant genetic loci that are associated with abnormal plasma levels of high-density lipoprotein (HDL) cholesterol, LDL cholesterol, total cholesterol or triacylglycerol (TAG) (Aulchenko et al., 2009; Kathiresan et al., 2009; Teslovich et al., 2010). Although GWA studies find candidate loci, mechanistic insights into how these polymorphisms lead to disease development and/or altered lipid metabolism usually requires extensive investigations in experimentally tractable whole-animal models.

Despite the established clinical importance of the management of serum lipids and a wealth of knowledge on lipoprotein synthesis and the cell biology of lipid metabolism, our understanding of whole-animal physiology remains far from complete. For example, most textbooks and review articles describe HDLs as the key pathway for returning excess cholesterol from tissues to the liver for excretion in the bile. This is why it is surprising that mice lacking the defining HDL protein (ApoA1) have normal biliary lipid secretion and bile acid metabolism (Amigo et al., 2011). This demonstrates that whole-animal studies will be best suited to elucidate the roles of specific genes in the etiology of dyslipidemias.

### Zebrafish as a model system for lipid and lipoprotein research

The zebrafish is remarkably similar to the human in its body plan, organ composition and physiology, and is easy to manipulate genetically (Fishman, 2001). Although characteristically used to study embryonic development, the zebrafish model is also contributing to marked advances in studies of dietary lipid absorption, obesity, diabetes and metabolic disease, fatty liver disease, lipoprotein biology and cardiovascular disease (reviewed in Anderson et al., 2011; Hölttä-Vuori et al., 2010; Seth et al., 2013). A recent review highlights the value of modeling dyslipidemias in zebrafish (Fang et al., 2014). In addition to studying lipid disorders in the context of whole-animal physiology, zebrafish researchers are identifying previously unappreciated roles for lipids and/or their metabolites in embryonic development (Avraham-Davidi et al., 2012; Cha et al., 2006; Chiang et al., 2011; Kai et al., 2008; Kawahara et al., 2009; Miyares et al., 2013; Schwend et al., 2011; Speirs et al., 2010; Zeng et al., 2009; Zhang et al., 2011). These studies are largely possible because of the many advantages of the zebrafish model system.

The fundamentals of lipid and lipoprotein metabolism are conserved among vertebrates. Like mammals, fish have three general lipoprotein pathways (Sire et al., 1981; Skinner and Rogie, 1978): (1) the exogenous lipoprotein pathway, where dietary lipids are packaged into chylomicrons (CM) for delivery to the tissues; (2) the endogenous lipoprotein transport pathway, where lipids that have been synthesized in the liver are delivered to the periphery by very-low-density lipoproteins (VLDLs), which are subsequently modified to ultimately yield LDL; and (3) the reverse cholesterol transport pathway, where HDL removes cholesterol from the tissues for excretion. Lipoproteins are composed of a core of neutral lipids, such as TAG and cholesterol ester (CE), surrounded by an envelope that contains phospholipids (predominantly phosphatidylcholine; PC), cholesterol and proteins that serve as receptor ligands and cofactors for enzymes. Notably, the lipoproteins isolated from the rainbow trout have a similar lipid and protein composition to human lipoproteins (Babin and Vernier, 1989). Zebrafish express the major classes of apolipoproteins (apoA, apoB, apoC, and apoE) and lipoprotein receptors (LDL receptor, *ldlr*; VLDL receptor, *vldlr*; and the major HDL scavenger, *scarb1*), as well as enzymes that load lipids into lipoproteins (such as microsomal triglyceride transfer protein, *mtp*; acyl-CoA:cholesterol acyltransferase, *acat*; and lecithin:cholesterol acyltransferase, *lcat*) and enzymes that release and/or exchange lipids during circulation (lipoprotein lipase, *lpl*; and cholesterol ester transfer protein, *cetp*) (Babin et al., 1997; Imai et al., 2012; Kim et al., 2011; Levi et al., 2012; Marza et al., 2005; Pickart et al., 2006; Thisse et al., 2004; Zhang et al., 2011).

**RESOURCE IMPACT**

**Background**

Abnormal serum lipid levels (dyslipidemias) are important risk factors for cardiovascular and other atherosclerotic diseases. Drugs that normalize lipid abnormalities are essential in the treatment of these diseases. However, many lipid disorders are complex and resistant to simple treatment strategies. Although diet certainly contributes to the development of dyslipidemias, genetic factors are also involved. Because lipid disorders are under complex genetic and physiological regulation, a thorough understanding of these mechanisms is essential for developing improved therapies. Thus, researchers are turning to animal models that share genetic and physiological traits with humans but that can also be manipulated genetically. Although the mouse model system is commonly used to understand lipoprotein metabolism, murine lipid physiology has some significant differences from humans. For example, cholesterol ester transfer protein, which is crucial for cholesterol transfer between different classes of lipoprotein, is lacking in mice but present in zebrafish. Furthermore, zebrafish are easy to manipulate genetically, share the fundamentals of lipid and lipoprotein biology with humans, and are emerging as a notable model system for studying lipid metabolism.

**Results**

In this study, the authors describe the value of the yolk-metabolizing stages of the zebrafish as a model for studying lipid and lipoprotein metabolism. The authors inject fatty acid tracers into the yolk of living zebrafish embryos and larvae, which enable them to follow the fate of fatty acids as they are metabolized, packaged into lipoproteins, excreted into the circulation and absorbed by the tissues of the body. Using a genetic model for abetalipoproteinemia, the authors show that secretion of labeled fatty acids into the circulation is dependent on lipoprotein production. Additionally, they examine the metabolic fate of exogenously delivered fatty acids by assaying their incorporation into complex lipids. Finally, they demonstrate that using this technique, they can detect lipid and/or lipoprotein abnormalities that are caused by a genetic mutation (in the microsomal triglyceride transfer protein) and by an inhibitor of a lipid-metabolizing enzyme (acyl-CoA:cholesterol acyltransferase).

**Implications and future directions**

These findings suggest that zebrafish yolk lipid transport and metabolism is a tractable model in which to study lipid and lipoprotein biology, regulation and metabolism. Zebrafish at these early stages are relatively simple, lacking many features that complicate the interpretation of metabolic studies. Notably, this readout of physiology can be readily combined with pharmacological and/or genetic tools to study the fundamentals of lipid metabolism. By studying lipid transport prior to the development of the digestive organs this technique might gain insight into genes that, when disrupted, cause developmental defects or larval lethality.

### Embryonic and lecithotrophic lipid metabolism

We believe that studying lipid and lipoprotein metabolism in the lecithotrophic, or yolk-metabolizing, stages of the zebrafish will provide fundamental insights into the cell biology and physiology of lipid metabolism. Assaying lipid and lipoprotein metabolism in these early developmental stages might even have some advantages over using free-feeding larva. Firstly, genes involved in lipid and lipoprotein metabolism are frequently expressed early in embryonic development where they can be crucial for early metabolic processes, proper embryonic development and/or viability (reviewed in Willnow et al., 2007). Indeed, in the zebrafish, many of the same genes that are important for lipid metabolism in the liver and intestine also process yolk lipids (described below). Genetic screens for mutants that have intestinal metabolism defects require that the animals survive to 6 or 7 days post-fertilization (dpf) and have proper intestinal development (Farber et al., 2001; Ho et al., 2006; Sadler et al., 2005; Watanabe et al., 2004). Mutants that have severe defects in yolk absorption would not meet these criteria. Secondly, unlike free-feeding larvae, the lecithotrophic stages are amenable to gene targeting by antisense morpholino oligonucleotides (MOs) (Heasman, 2002; Nasevicius and Ekker, 2000). MOs have been a beneficial tool to the zebrafish community to assess gene function, but their activity is usually limited to the first 3 dpf (Nasevicius and Ekker, 2000). An additional advantage offered by studying yolk lipid transport is the optical clarity and relative simplicity of the physiology when compared with later stages of development.

During early embryonic development, placental mammals utilize the visceral yolk sac, and later the placenta, for the uptake of maternal nutrients (Burton et al., 2001; Zohn and Sarkar, 2010), whereas oviparous vertebrates uptake nutrients from maternally deposited yolk (lecithotrophy) (Lange, 1981; Speake et al., 1998). Yolk proteins (complex phospho-lipo-glycoproteins, which bind hormones, vitamins and ions) are packaged inside specialized organelles called yolk granules (Opresko et al., 1980; Wallace et al., 1983). Yolk granules are modified lysosomes that store the yolk proteins until the nutrients are needed, and then the granules aid in the degradation of the yolk proteins (Fagotto, 1995). Oviparous organisms that undergo holoblastic cleavage, such as *Xenopus*, have yolk granules dispersed throughout the cells of the embryo (Herkovits and Ubbels, 1979; Jorgensen et al., 2009; Selman and Pawsey, 1965). By contrast, zebrafish embryos undergo discoidal meroblastic cleavage, which separates the developing embryo from the yolk (Kimmel and Law, 1985a; Kimmel and Law, 1985b), similar to an amniotic egg. Within the zebrafish yolk cell, surrounding the core of yolk granules is the yolk syncytial layer (YSL), a cytoplasmic layer that contains a syncytium of nuclei and a full complement of secretory machinery (Walzer and Schonenberger, 1979).

The YSL aids in the uptake of yolk nutrients in embryonic stages and during lecithotrophic larval stages. The YSL expresses many of the genes that are important for lipid metabolism and lipoprotein production. Genes expressed in the YSL include those that encode a majority of acyl-CoA synthetases (R.L.M. and S.A.F., unpublished; Thisse et al., 2004), fatty acid binding proteins (Mudumana et al., 2004; Sharma et al., 2004), the cholesterol transporter Abca1b (Thisse et al., 2004), apolipoproteins (Babin et al., 1997; Pickart et al., 2006; Thisse et al., 2004; Zhang et al., 2011), Mtp (Marza et al., 2005), the HDL scavenger Scarb1 (Thisse et al., 2004) and the nuclear receptors HNF4α, HNF4β and SHP-A (Bertrand et al., 2007). VLDL is secreted from the YSL once the circulatory system has formed (Mani-Ponset et al., 1996; Poupard et al., 2000; Walzer and Schonenberger, 1979). Lipoprotein production by the YSL is essential for yolk lipid utilization, as the yolk is not resorbed when *apoC2* or *mtp* are targeted by MOs (Pickart et al., 2006; Schlegel and Stainier, 2006) or when *mtp* is mutated (Avraham-Davidi et al., 2012). Similarly, the mouse yolk sac (Shi and Heath, 1984; Terasawa et al., 1999) and human placenta (Madsen et al., 2004) play important roles in embryonic lipoprotein synthesis and secretion. For example, mice lacking either ApoB (Farese et al., 1996) or MTP (Raabe et al., 1998) cannot export lipoproteins from the visceral yolk sac and thus die during mid-gestation.

Here, we describe a simple system to study lipid and lipoprotein biology using the lecithotrophic zebrafish. We deliver labeled (fluorescent and radioactive) fatty acids by using an oil-based microinjection into the zebrafish yolk, exploiting the properties of the YSL to efficiently metabolize lipids and generate lipoproteins. The fatty acid tracers allow us to visualize lipid and lipoprotein dynamics and to measure fatty acid metabolism. Furthermore, this method is also suitable for embryological studies. Our data reveal some potential changes in lipid metabolism over the course of development. Moreover, this technique is sensitive enough for both genetic and pharmacological studies. To complement this method, we characterized the endogenous lipid composition of larval zebrafish at a stage when yolk lipids are depleted, giving a glimpse of the complex lipids that are, ultimately, generated from the yolk.

## RESULTS

### A method to assay yolk lipid metabolism

**Fig. 1.**
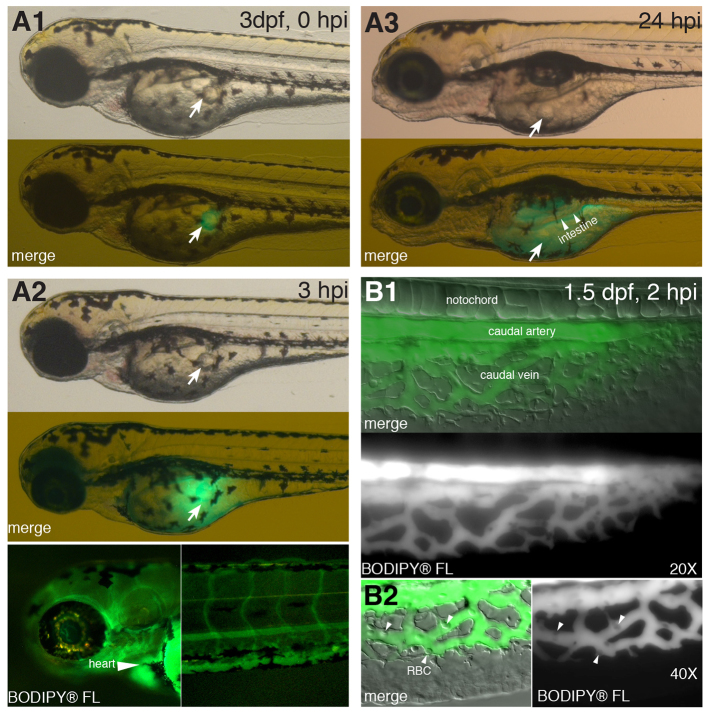
**Rapid absorption following yolk delivery of labeled fatty acid.** BODIPY-labeled fatty acid (BODIPY^®^ FL C_12_), dissolved in canola oil (0.5–1.5 ng/nl), was injected into the yolk of zebrafish larvae. (A1–A3) Timecourse of fatty acid absorption. BODIPY^®^ FL C_12_ (0.5 ng/nl) was injected into larvae at 3 dpf. The images shown are representative (*n*=4) and are taken from the same larva at different timepoints after injection (indicated in the top right-hand corner). Brightfield images in the top panels show the location of the canola oil drop (white arrow). The second panel shows combined brightfield and BODIPY^®^ FL C_12_ fluorescence (green) images. (A1) At 5–10 minutes post-injection. (A2) At 3 hpi. Bottom panels reveal BODIPY-labeled fatty acid in the heart (arrowhead) and head vessels (left) and tail vasculature (right). (A3) At 24 hpi. Bottom panel reveals BODIPY^®^ FL C_12_ in the yolk and newly formed intestinal lumen (arrowheads). (B) Labeled fatty acid enters the circulation. BODIPY^®^ FL C_12_ (1.5 ng/nl) was injected into 1.5-dpf larvae and analyzed 2 hours later. BODIPY-labeled fatty acid fluorescence is shown merged with Nomarski microscopy to reveal larval anatomy. Images shown are representative (*n*=4). BODIPY-labeled fatty acids were distributed in the caudal artery and the caudal vein. (B1) ×20 magnification. (B2) ×40 magnification. Arrowheads point to red blood cells (RBC).

To investigate lipid absorption and metabolism during zebrafish development, we delivered fluorescently and radioactively labeled fatty acids into the zebrafish yolk cell. Long- and very-long-chain fatty acids are hydrophobic and therefore pose substantial delivery issues when performing labeling studies in whole animals. We addressed this problem by concentrating and dissolving fluorescently labeled (BODIPY) or radiolabeled fatty acids in commercial canola oil. Canola oil is a TAG, composed of 62% oleic acid (lipid number C18:1, ω-9), 19% linoleic acid (C18:2, ω-6), 9% α-linolenic acid (C18:3, ω-3) and 7% saturated fatty acid (US Department of Agriculture National Nutrient Database, 2011). We microinjected the labeled fatty acid and oil mixture (2–4 nl) into the yolk of developing zebrafish (Fig. 1A). The embryo rapidly used the fluorescent and radiolabeled fatty acids. By contrast, the canola oil drop was not hydrolyzed until after the yolk had been absorbed, often remaining weeks after the injection (data not shown).

### Visualizing fatty acid uptake and transport

To assess fatty acid mobilization and transport, we followed a fluorescently labeled fatty acid analog after injection into the yolk of zebrafish larvae at 3 dpf. We chose a fatty acid that is covalently bound to the lipophilic BODIPY fluorophore, as BODIPY-labeled fluorescent fatty acid analogs closely resemble native fatty acids, with the BODIPY moiety adding the equivalent of approximately two to four carbon atoms to the acyl chain. Therefore, a 12-carbon fatty acid labeled with BODIPY (BODIPY^®^ FL C_12_) closely resembles palmitate (C16:0), the most abundant fatty acid in a majority of vertebrates, including fish (Tocher, 1995; Tocher et al., 2006).

Once injected, the BODIPY-labeled fatty acid analog (BODIPY^®^ FL C_12_, 0.5 mg/ml) rapidly diffused from the oil drop into the zebrafish yolk (Fig. 1A). Within 3 hours post-injection (hpi), fluorescence was visualized in the circulatory system (Fig. 1A2). By 24 hpi, the fluorescence was uniformly distributed throughout the yolk and it accumulated in the lumen of the newly formed intestine (Fig. 1A3). Additional analysis is needed in order to assess whether the primitive gut epithelium aids in the resorption of zebrafish yolk lipids, as has been shown in the sea bass (Garcia Hernandez et al., 2001). These data suggest that free fatty acid transport is extremely efficient and occurs independently of the metabolism of the TAG from the injected canola oil.

To further explore the use of this method to visualize lipid transport to the circulation, we altered the assay to enhance the fluorescent signal in the circulatory system. We accomplished this by choosing a stage of development that has a simple, easily observed circulatory system (1.5 dpf) and by increasing the concentration of BODIPY^®^ FL C_12_ to 1.5 mg/ml. At 2 hpi, fluorescence was readily observed in the caudal vasculature (Fig. 1B). The absence of fluorescence in the red blood cells (Fig. 1B2) indicates that the fluorescence is in the plasma.

### β-lipoproteins are essential for BODIPY^®^ FL C_12_ to enter circulation

For delivery to peripheral tissues via the circulatory system, lipids are packaged into plasma lipoproteins. To examine whether the yolk-delivered fatty acid analog was incorporated into lipoproteins, we examined a zebrafish model of abetalipoproteinemia (Avraham-Davidi et al., 2012), a disease characterized by the lack of ApoB-containing lipoproteins (β-lipoproteins, such as CM, VLDL and LDL). Mutations in the gene coding for MTP cause abetalipoproteinemia (Berthier et al., 2004; Sani et al., 2011; Wang and Hegele, 2000); therefore, we used a zebrafish model that has a hypomorphic mutation in *mtp* (Avraham-Davidi et al., 2012).

**Fig. 2.**
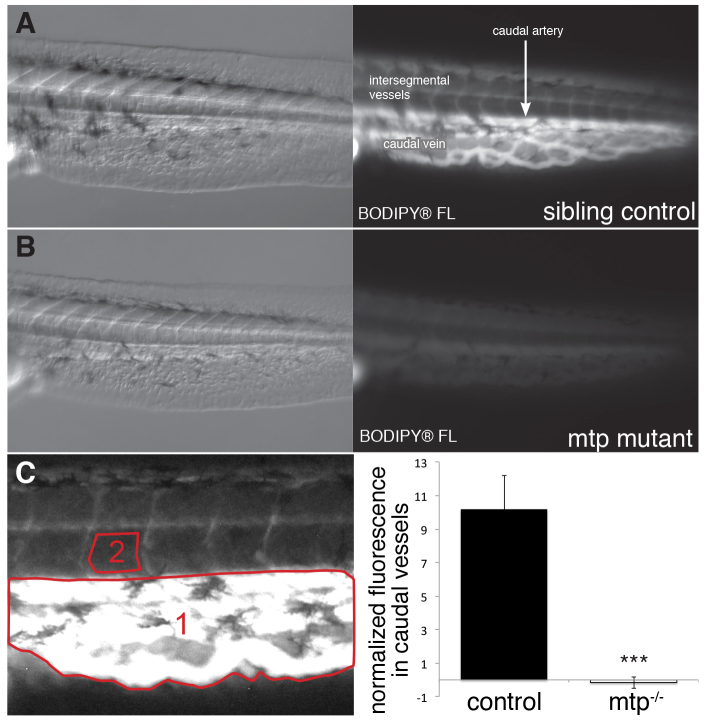
**Mtp mediates BODIPY^®^ FL C_12_ absorption.** BODIPY^®^ FL C12 (1.5 ng/nl) was injected into the yolk of 1.5-dpf larvae obtained from adult *mtp* heterozygote (*mtp*^+/−^) crosses. Following injection, larvae were incubated for 3 hours and then analyzed for vascular fluorescence. *mtp* mutants were identified phenotypically, as described previously (Avraham-Davidi et al., 2012). The images shown are representative for (A) wild-type and/or heterozygous siblings and (B) *mtp*-mutant larvae. Nomarski images are shown in the left panels. Fluorescent images (right panels) reveal that BODIPY^®^ FL C_12_ was distributed in the tail vasculature of wild-type and *mtp^+/−^* larvae (A), whereas *mtp*-mutant larvae (B) have negligible levels of vascular BODIPY-labeled fatty acid fluorescence. (C) Circulating BODIPY^®^ FL C_12_ fluorescence was quantified (shown in the right-hand panel) in the caudal artery and veins (area 1). A background correction was performed using a non-vascularized region (area 2). Four experiments were performed using 21–24 larvae per experiment. Data are represented as the mean of means±the pooled s.e.m., ***P<0.00001, Student’s *t*-test.

After the injection of BODIPY^®^ FL C_12_ into the yolk, no vascular BODIPY fluorescence was visible in larvae that were homozygous for the *mtp* mutation, whereas wild-type and heterozygous sibling larvae showed high levels of circulating BODIPY^®^ FL C_12_ (Fig. 2). These data are consistent with the incorporation of the yolk-delivered fatty acid tracer into β-lipoproteins in the YSL before they are secreted into the circulation. Although *mtp*-mutant fish have vascular defects (excess angiogenesis), caused by the lack of circulating ApoB-containing lipoproteins (Avraham-Davidi et al., 2012), our experiments were performed a day before the appearance of any vascular phenotypes.

### Metabolic fate of fatty acid tracer

**Fig. 3.**
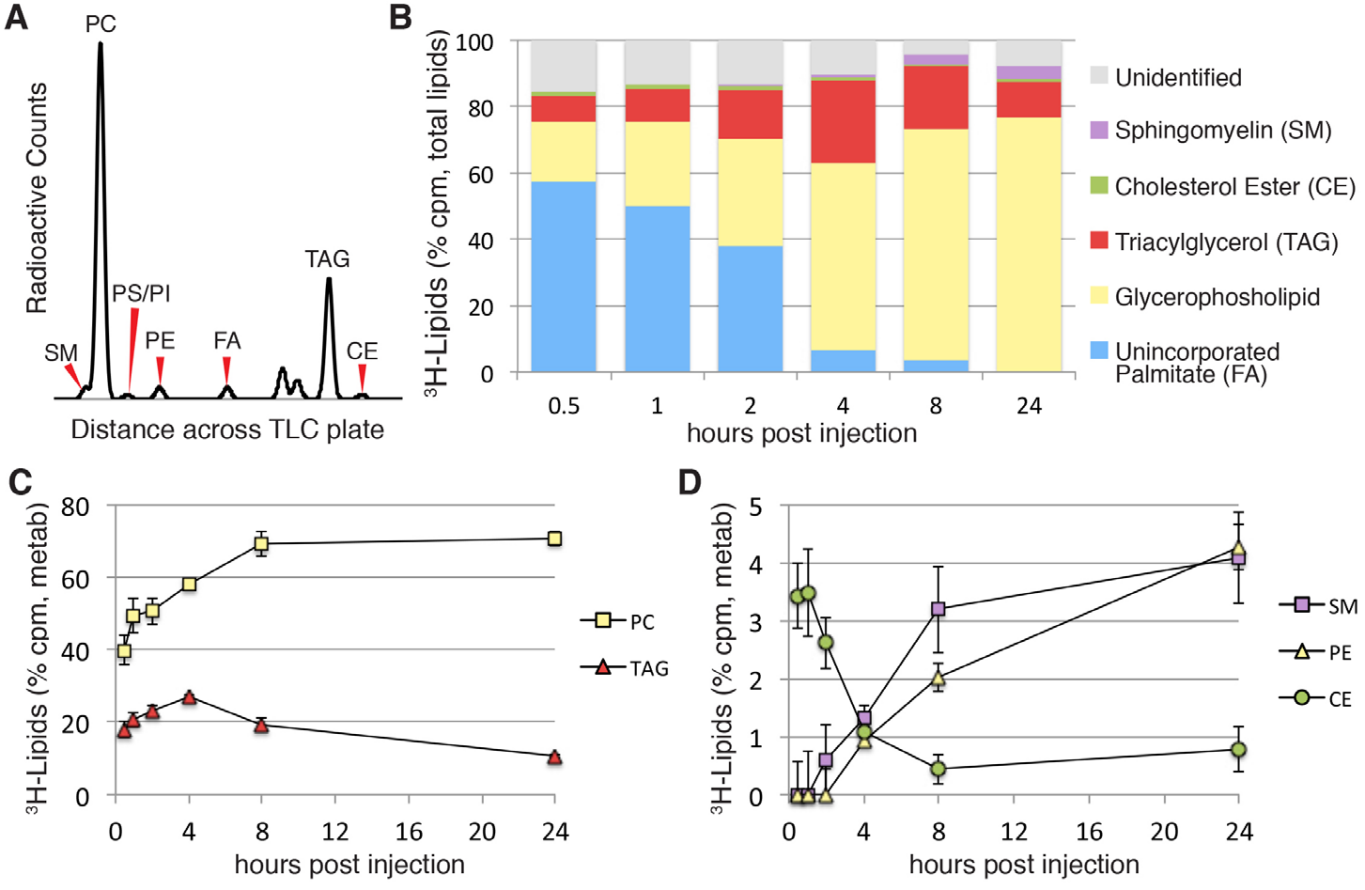
**Timecourse of palmitate absorption and incorporation.** ^3^H-palmitate (C16:0) was injected into the yolk of zebrafish larvae at 3 dpf. After incubation, the total larval lipids were extracted (15–20 pooled larvae) and separated by using TLC. PS and PI are reported together (PS/PI), and peaks that were not identified by using lipid standards were included in the analysis. Three or four experiments for each timepoint were performed. (A) A representative chromatograph showing radioactive peaks across a single lane of a TLC plate (8 hpi) is shown. Red arrowheads indicate lesser peaks present on the chromatographs. FA, fatty acid. (B) The proportional incorporation of ^3^H-palmitate into complex lipids over time. The mean percentages of the total counts (per minute; cpm) are shown. Total counts for individual glycerophospholipid peaks were grouped together and are reported as one value. (C,D) Timecourse of ^3^H-palmitate incorporation and utilization of radiolabeled neutral lipids and phospholipids. High (C) and low (D) abundance metabolites are shown on separate graphs. Data are represented as the mean±s.e.m. of radioactive metabolite (metab) counts (total counts minus unincorporated fatty acid).

We wanted to create a simple method that would give mechanistic insight when dramatic changes in lipid mobilization or circulating lipoproteins occurred. Moreover, alterations in lipid metabolism might not always lead to dramatic changes that can be readily visualized with BODIPY^®^ FL C_12_. To address both scenarios, we analyzed the metabolic fate of yolk-delivered radiolabeled fatty acids. Although BODIPY-labeled fatty acid analogs can be extremely useful for visualizing lipid absorption and transport in live zebrafish (reviewed in Otis and Farber, 2013), we chose to follow the metabolic fate of radiolabeled fatty acids because they can be accurately measured using a radioactive plate reader and do not require custom-made fluorescent lipid standards. We injected tritiated (^3^H) fatty acids into the yolk of embryos and lecithotrophic larvae. After a period of incubation, we extracted the lipids of pooled embryos or larvae, separated the different lipid classes using thin layer chromatography (TLC) and measured the abundance of radioactivity from each lipid class using a radioactive plate reader. A representative chromatograph is shown in Fig. 3A. The major peaks were identified by using lipid standards; however, some minor peaks were not.

### Timecourse of fatty acid metabolism

We aimed to test the hypothesis that radiolabeled fatty acids that had been delivered to the yolk in an oil drop would be readily metabolized into complex lipids. To this end, ^3^H-palmitate was injected into the yolk of 3-dpf larvae, and the incorporation of the labeled fatty acid into various lipid species was analyzed after 0.5, 1, 2, 4, 8 and 24 hours (Fig. 3; supplementary material Fig. S1). No appreciable loss of radioactivity was observed over the course of the experiment (data not shown), suggesting that minimal fatty acid oxidation occurred.

By 30 minutes post-injection, ~40% of the labeled fatty acid had been processed by the larvae (Fig. 3B; supplementary material Fig. S1A). In the first 2 hpi, ^3^H-palmitate was incorporated almost exclusively into PC, TAG and CE (Fig. 3; supplementary material Fig. S1), which are the major components of lipoprotein particles. By 4 hpi, over 90% of the ^3^H-palmitate had been incorporated into complex lipids (Fig. 3B; supplementary material Fig. S1D). In the circulatory system, LPL cleaves TAG from circulating lipoproteins, releasing fatty acids for cellular uptake and further metabolism (Arnault et al., 1996; Lindberg and Olivecrona, 2002; Wang et al., 2009). Supporting this, 4 hours after the injection, ^3^H-palmitate was incorporated into additional phospholipids other than PC [phosphatidlyethanolamine (PE), phosphatidylserine (PS), phosphatidylinositol (PI) and sphingomyelin (SM)] (Fig. 3D; supplementary material Fig. S1D–F). This timing corresponds well with our visualization of BODIPY fluorescence in the vasculature (Fig. 1). By 24 hpi, 100% of the radiolabel had been incorporated into complex lipids, with a majority (70.4±2.4%, ±s.e.m.) existing as PC and TAG (10.6±0.5%) (Fig. 3; supplementary material Fig. S1F).

### BODIPY^®^ FL C_12_ is metabolized in a manner similar to that of native fatty acids

Any study that uses fluorescent lipid analogs raises the concern that they are processed differently to native fatty acids. Indeed, there are mixed reports as to whether the BODIPY-labeled analogs are processed as native lipids (Boldyrev et al., 2007; Dahim et al., 2002; Furlong et al., 1995; Hendrickson et al., 1999; Hölttä-Vuori et al., 2008; Huang et al., 2002; Li et al., 2005). We therefore wanted to examine whether BODIPY^®^ FL C_12_ is metabolized in a manner similar to that of ^3^H-palmitate. Using BODIPY^®^ FL C_12_ at the molar equivalent to ^3^H-palmitate, we found that the majority of fatty acid incorporation was strikingly similar between the two labeled fatty acid analogs. For example, after 6 hpi, 65±8% (±s.e.m.) of the BODIPY^®^ FL C_12_ was incorporated into phospholipids and 25±5% into TAG and/or CE (Table 1), which is, approximately, what would be expected based on results with ^3^H-palmitate (Fig. 3). Given that a higher concentration of BODIPY^®^ FL C_12_ was injected in order to image fatty acid mobilization (Fig. 1A), we confirmed that the higher concentration of BODIPY^®^ FL C_12_ was also metabolized into complex lipids (Table 1).

**Table 1.**
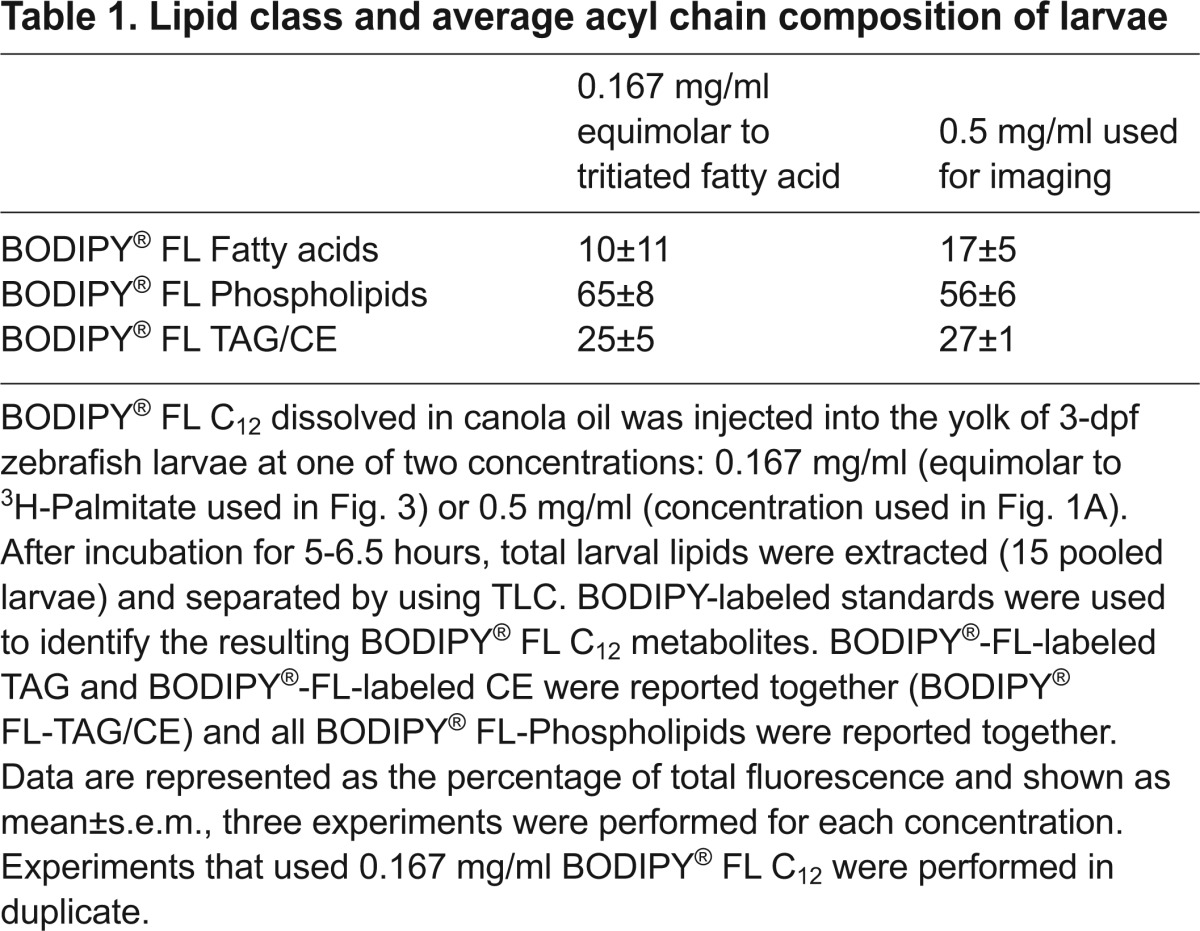
Lipid class and average acyl chain composition of larvae

After yolk injection of fluorescently labeled fatty acids, we observed the rapid appearance of BODIPY® FL C_12_ fluorescence within the circulation (Figs 1,2). This observation, coupled with the rapid metabolism of yolk-delivered radiolabeled fatty acids (Fig. 3), indicates that yolk injection is an efficient method for delivering fatty acid tracers for metabolic studies. Our approach, delivering labeled fatty acids to the yolk and assessing transport and/or metabolism, is an effective model for lipid metabolism and lipoprotein transport studies.

### Fatty acid composition of larval lipids

Eukaryotic cells have thousands of different lipid species, which are increasingly being appreciated by the wider life sciences community (Fahy et al., 2011; van Meer et al., 2008). One way in which this range of species is created is by acyl chain diversity. Different lipid classes are known to have preferences for specific acyl chains, and this can vary depending on species and tissue type; therefore, a comprehensive study of larval lipids is necessary in order to provide a context for metabolic studies in zebrafish.

In order to establish a baseline and resource for future experiments, we profiled the lipids of larval zebrafish at the stage when yolk stores are largely depleted, but before exogenous feeding begins (6 dpf). We chose a stage when zebrafish larvae are capable of consuming dietary lipids, so that the data might be useful for experiments analyzing either yolk lipid metabolism or dietary lipid metabolism. Monroig et al. characterized the total acyl chain profile of larvae at 3 dpf, but they did so irrespective of lipid class (Monroig et al., 2009). Thus, we partnered with a commercial vendor (Lipomics Technologies) to assess both the lipid class and acyl chain constituents. Each lipid class was separated by preparative TLC, and the acyl chain profile of each was determined (Table 2; supplementary material Table S1). Consistent with Monroig et al., we observed that C22:6 ω3 was the most abundant polyunsaturated fatty acid (PUFA) in larvae at 6 dpf. Further, the zebrafish TAG acyl chain profile (Table 2) was quite similar to that reported for white seabream (*Diplodus sargus*) free-swimming larvae, with the abundance of saturated fatty acids being about twice that of monounsaturated fatty acids or PUFAs (Cejas et al., 2004). ω3 fatty acids were significantly more abundant than ω6 fatty acids in the PC, PE, PC and TAG lipid classes (in contrast with cardiolipin, where ω6 fatty acids predominated).

**Table 2.**
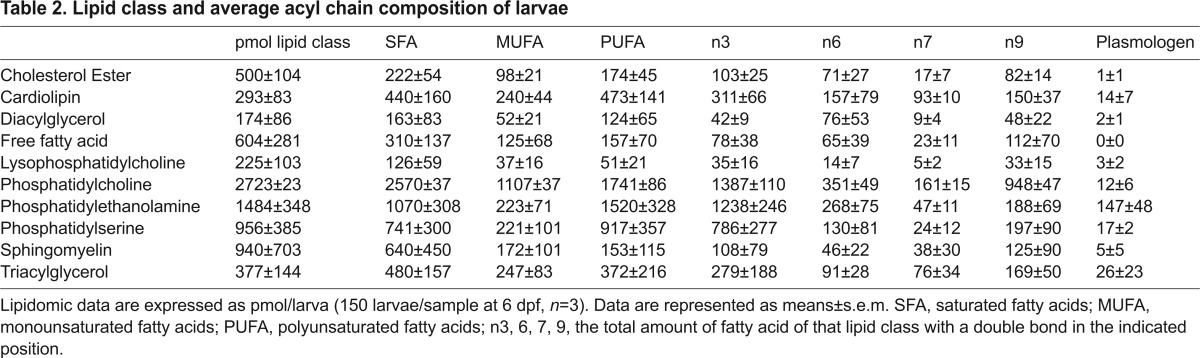
Lipid class and average acyl chain composition of larvae

### Differential fatty acid incorporation

**Fig. 4.**
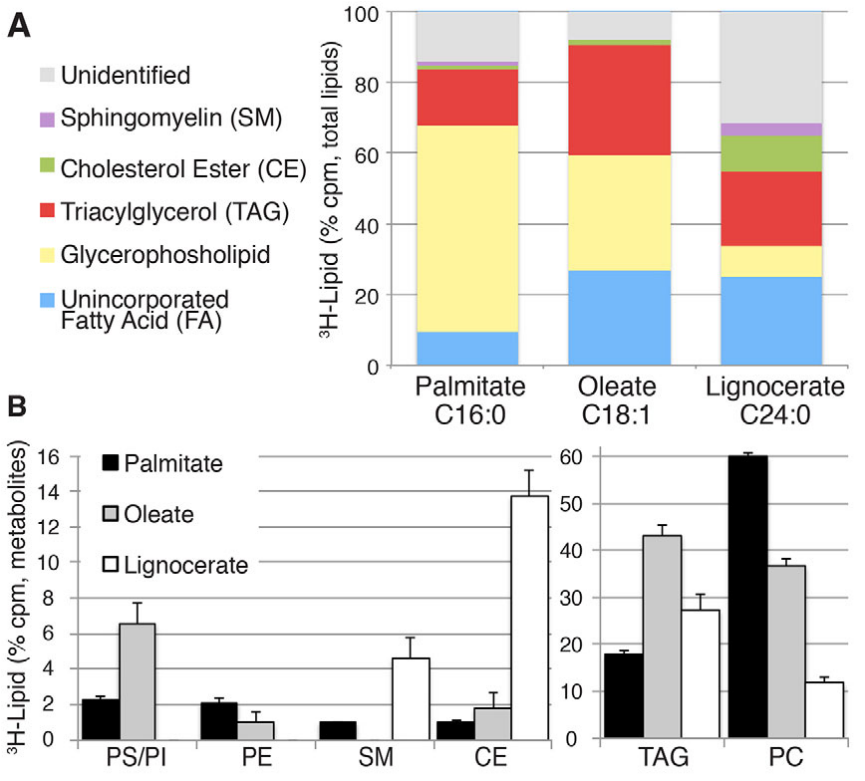
**Differential fatty acid absorption and incorporation in lecithotrophic larval zebrafish.** ^3^H-palmitate (C16:0), ^3^H-oleate (C18:1) or ^3^H-lignocerate (C24:0) was delivered into the yolk of larvae at 3 dpf. Following 3 hours of incubation, total larval lipids were extracted (15–20 pooled larvae) and analyzed as previously described in Fig. 3. Three or four experiments were performed for all analyses. (A) The proportional incorporation of ^3^H-fatty acids into complex lipids differs between the different fatty acids. The mean percentage of total counts is shown. The total counts for individual glycerophospholipid peaks were grouped together and are reported as one value. (B) Differential incorporation of radiolabeled fatty acids into neutral lipids and phospholipids. Data are represented as the mean±s.e.m. of radioactive metabolite counts. Lower (left) and higher (right) abundance metabolites are shown on separate graphs. A significant main effect of ^3^H-fatty acid species on complex lipid synthesis was observed (P<0.05, ANOVA), indicating that fatty acid type influences the rates of subsequent lipid synthesis. A comprehensive post-hoc analysis revealed significant differences between the percentage of labeled fatty acid incorporation within each class of lipids (REGWQ analyses; data not shown).

To examine whether we could ascertain similar differences to those observed above in acyl chain preferences using our labeling method, we compared the incorporation of three different tritiated fatty acid species in larvae at 3 dpf: a monounsaturated long-chain fatty acid, oleate (C18:1, ω9); a saturated long-chain fatty acid, palmitate (C16:0); and a saturated very long-chain fatty acid, lignocerate (C24:0) (Fig. 4). For a comparison with endogenous lipids, supplementary material Table S2 shows the relative abundance of these acyl chains in lipid species from larvae at 6 dpf.

We found that under the experimental conditions used here, palmitate was used more efficiently than either oleate or lignocerate (Fig. 4A). This was expected as palmitate is the most abundant endogenous fatty acid at this stage in zebrafish development (Monroig et al., 2009). Additionally, palmitate is a resident acyl chain in a majority of lipid classes in the 6-dpf larvae (supplementary material Table S1). Oleate incorporated into TAG and PS and PI more readily than the other fatty acids tested, whereas palmitate was most efficiently incorporated into PC (Fig. 3B). The very-long-chain fatty acid (lignocerate) was poorly incorporated into glycerophospholipids, both endogenously (supplementary material Table S1) and in our labeling study (Fig. 3A). By contrast, lignocerate had the highest incorporation into SM (Fig. 4B). Although endogenous CE in zebrafish has little lignocerate (supplementary material Table S1), a considerable percentage of yolk-delivered ^3^H-lignocerate was incorporated into CE (Fig. 3). Yolk-delivered fatty acids displayed differential incorporation into complex lipids. For the most part, these differences were comparable to the lipid preferences exhibited by endogenous lipid pools (supplementary material Table S2).

### Fatty acid metabolism during embryogenesis

**Fig. 5.**
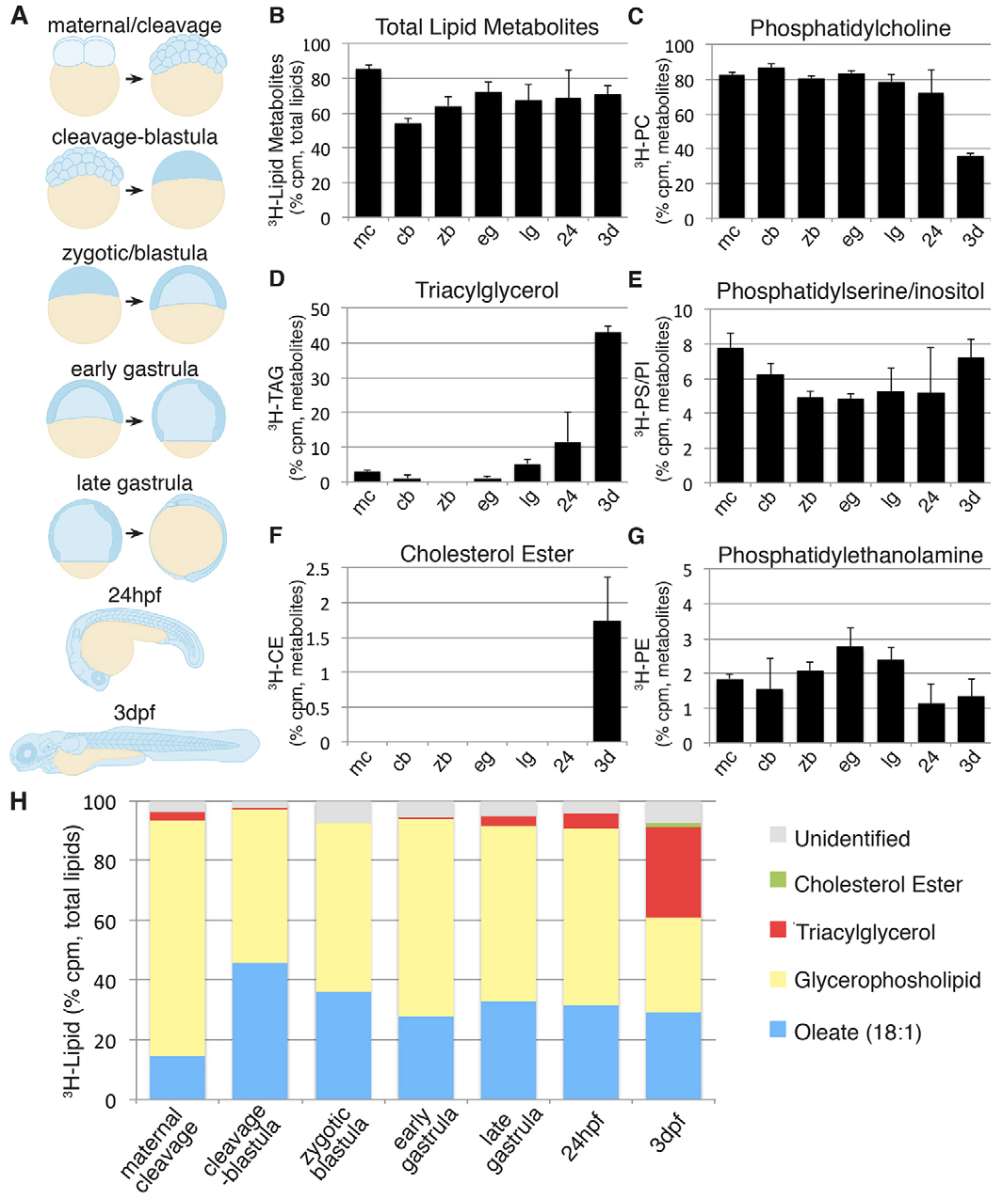
**Developmental analysis of fatty acid metabolism.** ^3^H-oleate was injected into the yolk at selected developmental timepoints. Following 2 hours of incubation, lipids were extracted (15–20 pooled embryos or larvae) and analyzed. Three or four experiments were performed for all analyses. (A) The following developmental stages were chosen. Maternal-cleavage (0.75 hpf; two-cell to 1000-cell stage): only maternally deposited mRNA exists. The yolk cytoplasm streams into the overlying embryo until 2 hpf (mc). Cleavage-blastula (2 hpf; 64-cell to sphere stage): the yolk and overlying embryo are separated by cell membranes. Zygotic genes are induced (~2.75 hpf) and the YSL forms at ~3 hpf (cb). Zygotic-blastula (oblong; 3.7 hpf to 50% epiboly): the zygotic genome is expressed, the blastula begins to be patterned and cell movements begin (zb). Early gastrula (5.3 hpf; 50% epiboly to 70% epiboly): gastrulation begins (eg). Late gastrula (8 hpf; 70–80% to bud): the YSL begins to express many lipid metabolism genes (lg). 24 hpf (24–26 hpf): embryonic patterning and somitogenesis are complete, the circulatory system begins to function (24). 3 dpf: lecithotrophic larvae with robust circulatory system and primitive intestine and liver (3d). (B–G) Quantification of total (B) and individual ^3^H-oleic acid metabolites (C–G) across the developmental stages. Data are normalized as the percentage of the total metabolites because of the variable amount of fatty acid incorporation and are shown as mean±s.e.m. (H) Proportional absorption and incorporation of ^3^H-oleate into complex lipids during zebrafish development. The mean percentage of total counts is shown. The total counts for individual glycerophospholipid peaks were grouped together and are reported as one value.

The externally developing zebrafish is an ideal system to explore roles for lipid metabolism in aspects of developmental biology. Therefore, we chose to examine the utility of our labeling approach at several stages of zebrafish development (Fig. 5; supplementary material Fig. S2). ^3^H-oleic acid was injected into the yolk of zebrafish embryos and larvae, which were staged according to Kimmel and colleagues (Kimmel et al., 1995). The fish were incubated for 2 hours after the injection in order to assess fatty acid metabolism in the stages that are described in Fig. 5A (from the two-to four-cell embryo to the lecithotrophic larva at 3 dpf).

For the ‘maternal-cleavage stage’, the fatty acid was injected between the 2- and 4-cell stage (0.75–1 hour), when the yolk cytoplasm streams into the overlying cells of the embryo. The fatty acids (free or bound to fatty acid binding proteins) can therefore diffuse directly into the cells, potentially explaining why the fatty acid substrate was metabolized so efficiently at this stage (Fig. 5). Maternal mRNAs and/or proteins, which are deposited during oogenesis, provide the enzymatic machinery to promote the incorporation of ^3^H-oleic acid into all of the glycerophospholipids and TAG (Fig. 5). After the initiation of zygotic transcription (~2.75 hpf), the developing embryo incorporates ^3^H-oleic acid primarily into glycerophospholipids until the end of gastrulation (8 hpf; Fig. 5). There was an increase in PE formation in the beginning of gastrulation (Fig. 5F); this points to a potential role for PE in cell specification or gastrulation movements – a new area to explore.

The change in metabolism at the late-gastrula stage coincides with the timing of zygotic expression of many lipid metabolism genes in the YSL (Babin et al., 1997; Marza et al., 2005; Sharma et al., 2004). A large increase in neutral lipid (TAG and CE) formation occurred at 3 dpf, when both the lipoprotein machinery is expressed in the YSL and the circulatory system is fully functional (Fig. 5A,C,E). Taken together, these data demonstrate clear variations in fatty acid metabolism throughout embryonic development. Considerably more research is required in order to determine the potential relevance of these differences.

### Validation of the method by using drug treatment

The radioactive fatty acid incorporation analyses were all performed on wild-type zebrafish. Although this provides a foundation for understanding lipid metabolism during the embryonic and larval stages of zebrafish development, we wanted to confirm that this methodology could also detect alterations in enzymatic activity. Therefore, we treated larvae (at 3 dpf) with an inhibitor of acyl-CoA:cholesterol acyltransferase (ACAT), the acyltransferase that is used to synthesize CE from cholesterol and fatty acid. ACAT activity is also important in the production of β-lipoproteins (Rudel et al., 2001). We performed this experiment with lignocerate, as it was more readily incorporated into CE than the other fatty acids tested (Fig. 4).

**Fig. 6.**
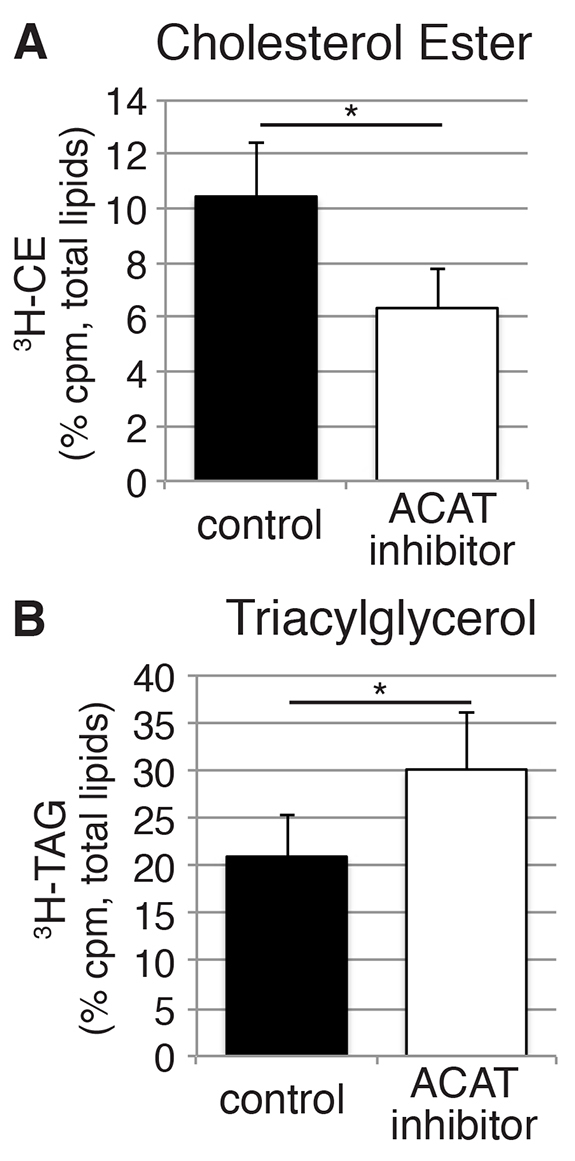
**The assay is sensitive to alterations in enzyme activity.** At 3 dpf, larvae were pretreated with 200 μM ACAT inhibitor (CAY10486) or vehicle for 2 hours before injection of ^3^H-lignoceric acid into the yolk, larvae were then incubated in ACAT inhibitor or vehicle for a further 4 hours after injection. Lipids were extracted (15–20 pooled larvae) and analyzed as described previously in Fig. 3. Data are represented as the percentage of the total counts and are shown as mean±s.e.m., *n*=3, *P>0.05, Student’s *t*-test. (A) Proportional incorporation of ^3^H-lignoceric acid into CE. (B) Proportional incorporation of ^3^H-lignoceric acid into TAG.

As anticipated, larvae that had been treated with an ACAT inhibitor (CAY10486, 200μM) exhibited a decreased incorporation of lignocerate into CE compared with larvae that had been treated with the vehicle (*P*<0.05; Fig. 6A). Interestingly, the percentage of unincorporated ^3^H-lignocerate did not significantly change (control 25±7%, ACAT inhibitor 20±5%, ±s.e.m.); rather, the percentage of TAG increased ~10% (*P*<0.05; Fig. 6B). These data highlight the potential of this technique to be used to evaluate new pharmacological modulators of lipid metabolism.

## DISCUSSION

Here, we describe an experimental model that can be used to investigate lipid and lipoprotein metabolism. We utilized both fluorescent and radiolabeled fatty acids to obtain a spatiotemporal understanding of zebrafish yolk lipid metabolism. These labeled fatty acids act as tracers that enable us to follow their metabolic fate as fatty acids are packaged into lipoproteins, excreted into the circulation and absorbed by the tissues of the body. Furthermore, we demonstrate that this method is sensitive to changes in enzymatic activity (Fig. 6) and genetic manipulations (Fig. 2).

### Yolk lipid processing

We believe that complex lipids present in the yolk are first hydrolyzed within the yolk granule (a specialized lysosome-like organelle) to release fatty acids that are rapidly re-synthesized into TAG, CE and PC. These newly synthesized lipids are incorporated into nascent lipoprotein particles. The YSL is implicated in this early step of yolk lipid metabolism, as it expresses many genes that are involved in lipid metabolism and lipoprotein biogenesis and because YSL secretion of VLDL particles has been described in other teleost species (Mani-Ponset et al., 1996; Poupard et al., 2000; Walzer and Schonenberger, 1979). Our data supports this model of early lipoprotein-mediated yolk lipid transport because *mtp* mutant fish (which cannot produce β-lipoproteins, such as VLDL) do not acquire vascular fluorescence after injection with BODIPY-labeled fatty acids (Fig. 2). Moreover, the lipids that were initially formed (0.5–2 hours) post-injection of radiolabeled fatty acids are all well-known components of circulating lipoproteins (Fig. 3; supplementary material Fig. S1A–C). The formation of additional phospholipids (SM, PS, PI and PE) at later timepoints (Fig. 3; supplementary material Fig. S1D–F) might reflect the secondary metabolism of lipoprotein-derived fatty acids, after lipoproteins that have been excreted from the YSL are hydrolyzed in peripheral tissues by endothelial hydrolases.

### A canola oil delivery system

Lipophilic compounds represent a class of bioactive molecules that pose unique challenges for solubilization and delivery to tissues and cells in aqueous environments. Nature has solved this challenge with the evolution of lipoproteins. Our data suggest that a canola oil drop delivery system is an extremely efficient approach to globally distribute fatty-acid-containing lipids through the YSL lipoprotein transport machinery. Although we have only examined fatty acid analogs, we believe this will serve as an efficient delivery system for other hydrophobic molecules. We expect that the biophysical properties of the desired hydrophobic cargo will greatly influence the efficiency of the transport and metabolism.

Liberation of labeled fatty acids analogs from the canola oil drop probably occurs by a process that differs from fatty acid hydrolysis from endogenous yolk proteins. Yolk proteins are packaged into yolk granules, which aid in their gradual hydrolysis in order to nourish the developing embryo (Jaroszewska and Dabrowski, 2009). Feedback mechanisms regarding when and how yolk granules liberate the packaged lipids are not well described. Because we do not deliver the canola oil drop directly into the yolk granules, we believe that the fatty acid analogs released from the canola oil are treated as post-lysosomal metabolites.

Regarding the metabolism of the TAG of the canola oil drop, we hypothesize that it is treated in a similar manner to a naturally occurring oil globule; other teleosts (such as the goldfish and turbot) segregate maternally contributed neutral lipids into a large oil globule that is utilized after yolk absorption (Poupard et al., 2000; Wiegand, 1996).

### Advantages of the lecithotrophic zebrafish for studying lipid and lipoprotein biology

There are a number of animal models where genetic mutations and/or dietary changes can lead to dyslipidemia (de Winther and Hofker, 2002; Fang et al., 2014; Moghadasian et al., 2001; Russell and Proctor, 2006). Although each model system has advantages and disadvantages, the mouse and zebrafish model systems stand out for their amenability to combine physiology and genetic studies. One notable difference between these two genetic models is the presence of Cetp in zebrafish (Kim et al., 2011; Thisse et al., 2004), an enzyme that is important in the transfer of cholesterol between different classes of lipoproteins and a therapeutic target in humans (Bochem et al., 2013). Studying lipoprotein levels and composition in the mouse often requires drawing blood and measuring serum lipid levels. Although informative, these levels reflect a snapshot of steady-state lipoprotein metabolism, reflecting the activities of processes ongoing in a variety of tissues, and it might be difficult to discern the underlying mechanisms for any differences observed. Utilizing lipid tracers in lecithotrophic zebrafish larvae has the distinct advantage of being able to discriminate between alterations in lipoprotein biogenesis and the subsequent metabolism.

We propose that the rapidly developing embryonic and lecithotrophic larval zebrafish is a vectoral lipid transport system, whereby lipids are mobilized, processed and shipped to developing tissues. Early-stage zebrafish lack adipose depots and a highly functional liver, do not eat exogenous food and are likely to be relatively free of the influence of microorganisms. In short, they lack features that are known to complicate the interpretation of studies performed at later stages. Despite this deficit, evidence suggests that the fundamental regulatory processes at the whole-organ and single-cell levels remain intact.

### Future studies

The fluorescent and radioactive methods can be used separately or in combination to elucidate different aspects of lipid and lipoprotein biology, and potential roles in development. The fluorescent method might prove to be useful for examining lipoprotein dynamics. In fish that lack β-lipoproteins, we see a complete loss of vascular fluorescence (Fig. 2). Thus, it might be possible to assay for decreased or elevated levels of lipoprotein synthesis. Additionally, the rate and/or efficiency of fluorescently labeled lipid clearance from the circulation could be monitored to look for factors that influence lipoprotein dynamics.

Combining the approach that we have described with genetic tools, such as fluorescent transgenes, antisense MOs, mRNA expression, targeted mutations or mutagenic screens, would be beneficial for examining the etiology of dyslipidemias. In addition, it would be possible to study the influence of genes that, when mutated, result in larval lethality – e.g. as a result of defects in the development of the liver and/or intestine – because the approach can be performed at much earlier stages during embryonic development. The described BODIPY^®^ FL C_12_ oil drop delivery system could be used for a fairly high-throughput forward genetic screen. By contrast, the delivery of radiolabeled fatty acids via an oil drop is not amenable to a primary screen because the subsequent TLC assay requires lipid extracts derived from multiple injected embryos or larvae. However, it would be a powerful secondary assay for mutant characterization. The data generated thus far demonstrates that this approach is a viable strategy for understanding the physiological basis of mutations or for examining candidate genes using reverse genetic tools.

## Conclusion

Here, we describe a method for accurately measuring yolk-delivered fatty acid uptake and metabolism in the zebrafish. We complement this description with a detailed analysis of endogenous larval lipids. We illustrate the utility of this technique throughout the lecithotrophic stages of zebrafish development (0–3 dpf). Moreover, we demonstrate that this method is sensitive to changes in enzymatic activity and in a dyslipidemia disease model. There is a body of literature highlighting the many similarities between fish and mammalian lipid metabolism, which serves to increase the utility of this powerful model vertebrate. Our data suggest that zebrafish yolk lipid transport and metabolism is a tractable model to study lipoprotein biology, regulation and metabolism. This readout of physiology can be readily combined with pharmacological and/or genetic tools to study the fundamentals of lipid metabolism.

## MATERIALS AND METHODS

### Fish husbandry

Wild-type embryos were obtained from FWT intercrosses. The FWT strain was generated from the AB strain outcrossed once to a wild-type strain from a commercial supplier (to reintroduce hybrid vigor) and then inbred several generations. Embryos were collected from natural spawning and raised and staged as described previously (Kimmel et al., 1995). The *mtp* mutant strain *stalactite* was generously provided by Karina Yaniv and Brant Weinstein (Avraham-Davidi et al., 2012). *mtp* mutants were identified by the characteristic darkened yolk. Zebrafish care and experimental procedures were performed in accordance with the Animal Care and Use Committees of the Carnegie Institution animal protocol no. 139, ‘Lipid signaling during zebrafish development’.

### Labeled fatty acid injection into yolk

To analyze lipid metabolism in zebrafish embryos and lecithotrophic larvae, we injected fluorescently tagged and radiolabeled fatty acid analogs into the yolk. BODIPY-labeled (BODIPY^®^ FL C_12_; Invitrogen D3822) and tritiated fatty acids (lignoceric acid [C24:0-ART 0865], oleic acid [C18:1-ART 0198] and palmitic acid [C16:0-ART 0129]; American Radiolabeled Chemicals) were dried down completely (from their storage solutions) in a speed vacuum and re-suspended in canola oil (Radiolabeled fatty acids: 16.77 μCi/μl, BODIPY^®^ FL C_12_: 0.5–1.5 μg/μl). Oil (3–5nl) was injected into the zebrafish yolk (roughly 20,000 counts per minute/embryo) with a microforged glass needle (P-97 Flaming/Brown micropipette puller, Sutter Instruments) connected to a N_2_ gas pressure injector (PLI 100, Harvard Apparatus). The tips of the injection needles were broken with forceps to increase the size of the bore to allow for oil filling. The labeled fatty acid in oil was pipetted into glass capillaries secured on a stereomicroscope stage. Injection needles were inserted into the capillaries and loaded by applying suction from a 50 cc syringe. A stereomicroscope was employed throughout the loading process to ensure that the needle tip remained in the oil and was not broken. Fish, 24 hpf and older, were anesthetized with 0.03% tricaine and washed immediately after injection in warmed embryo media. Embryos at early stages were injected vegetally, avoiding the embryo proper. Embryos at later stages (≥24 hpf) were injected ventrally and posteriorly, avoiding penetrating the body of the embryo or larva, as well as sections of the yolk that have overlying vasculature. Embryos and larvae were incubated in embryo media at 28.5°C for 0.5 to 24 hours. For lipid extraction, 10–20 embryos or larvae were pooled. Before homogenization for lipid analysis, embryos and larvae were inspected to confirm the presence of an oil drop (see Fig. 2). Any fish (wild type, mutant or treated with drug) that did not appear normal or healthy due to developmental abnormalities, anesthesia or trauma from the oil injection were not included in the studies. Heartbeat, blood circulation and the ability to swim were assessed in larvae at 3 dpf.

### Drug treatment

Two hours before fatty acid injection, 3-dpf larvae were soaked in a 200 μM solution of ACAT inhibitor (Cayman Chemical, CAY10486) or dimethylsulfoxide vehicle. After fatty acid injection, the larvae were incubated in fresh ACAT inhibitor or vehicle for 4 hours at 28.5°C.

### Lipid extraction

Total lipids were extracted according to the Bligh and Dyer method (Bligh and Dyer, 1959). For lipid extraction, 10–20 embryos or larvae were pooled. Before homogenization for lipid analysis, oil-injected zebrafish were inspected to confirm survival and presence of an oil drop (see Fig. 1A). Zebrafish were transferred to a microfuge tube in minimal media and 200 μl of homogenization buffer (20 mM Tris, 1 mM EDTA) was added. The embryos or larvae were then homogenized by hand using a mini-pestle and 750 μl of chloroform:methanol (1:2) was added to the homogenate, followed by 250 μl of chloroform and 250 μl of homogenization buffer. Samples were vortexed for 30 seconds after the addition of each extraction reagent. Samples were incubated at ~25°C for ~5–10 minutes and centrifuged at 3000 ***g*** for 5 minutes. The organic phase was transferred to a clean microfuge tube and stored at −80°C for TLC analysis.

### Thin layer chromatography

Lipid samples were dried down in a speed vacuum to ~5 μl. Samples were resuspended in 40 μl chloroform:methanol (2:1) and loaded onto silica gel chromatography plates (LK5D, Whatman). A two-solvent system was used to separate the lipid metabolites. The polar solvent (ethanol:triethylamine:water; 27:25:6.4) was run first, halfway-up the plate. The plate was allowed to air dry before running the non-polar solvent (petroleum ether:ethyl ether:acetic acid; 64:8:0.8) to near the top of the plate. Standards were run in adjacent lanes. Unlabeled standards were used for the phospholipids PC, PS, PI, PE and SM, as well as the neutral lipids TAG and CE to identify the resulting radioactive lipid peaks. To locate the migration of lipid standards, TLC plates were sprayed with chromic-sulfuric acid (5% w/v potassium dichromate solution in 40% v/v aqueous sulfuric acid solution) and placed on a hot plate (>180°C) to char lipids. TLC analysis of the BODIPY^®^-FL-labeled samples was processed using only the non-polar solvent and BODIPY^®^-FL-labeled standards, as described previously (Carten et al., 2011).

### Detection of radiolabeled lipids

Dried TLC plates were scanned for total counts across all lanes using a Bioscan radio-TLC Imaging Scanner (Bioscan, AR-2000). The scanner uses a gas-filled proportional counter to directly count tritium’s β emission across each lane of a TLC plate. Background counts were subtracted from all total lane counts before peak analysis.

### Peak analysis

Data obtained from the Imaging Scanner was exported from the Winscan software package for more detailed analysis. The Peak Analyzer Pro software package (8.6, OriginLab) was used to determine and subtract baselines, as well as to find, integrate and fit peaks with a Gaussian distribution. The software package discriminates peaks that partially overlap, enabling analysis of all metabolites of the ^3^H-fatty acids. Data are represented as chromatograms or graphed as percent of total counts or total metabolites (total counts, not including the unincorporated fatty acid peak).

### Detection of BODIPY^®^-FL-labeled lipids

Dried TLC plates were scanned (Typhoon Scanner, GE Healthcare) using the blue fluorescence laser (excitation, 450 nm; emission, 520 nm band-pass filter) to detect fluorescent bands that were quantified using ImageQuant software (GE Healthcare). Control lanes of uninjected larvae were used for background correction to account for any naturally fluorescent lipids.

### Measurement of BODIPY^®^-FL-labeled circulating lipoproteins

ImageJ was used to quantify BODIPY fluorescence in the caudal artery and caudal vein after the injection of BODIPY^®^ FL C_12_ into the yolk. Background (a small non-vascularized area of the fish between the intersegmental vessels) fluorescence was subtracted from the measurement. In each experiment, the exposure was kept constant.

### Acyl chain analysis of endogenous larval lipids

At 6 dpf, zebrafish larvae (150 larvae/sample) that had not been given exogenous food were snap frozen and shipped to Lipomics Technologies (West Sacramento, CA) for acyl chain profiling of individual lipid classes that were separated by preparative TLC (Watkins et al., 2002). Each class of lipid was quantified and then trans-esterified (3 N methanolic-HCl) to fatty acid methyl esters that were then measured by gas chromatography. Lipidomic data were calculated as pmol/larva from three complete data sets.

## Supplementary Material

Supplementary Material
