# Supplementary material for: Zebrafish yolk lipid processing: a tractable tool for the study of vertebrate lipid transport and metabolism
Source: Dis Model Mech. 2014 May 8;7(7):915–27. doi: 10.1242/dmm.015800 (PMC4073280; doi:10.1242/dmm.015800)
Supplement: Supplementary Material [file supp_7_7_915__index.html]

Zebrafish yolk lipid processing: a tractable tool for the study of vertebrate lipid transport and metabolism — Supplementary Material 

# Zebrafish yolk lipid processing: a tractable tool for the study of vertebrate lipid transport and metabolism

## DMM015800 Supplementary Material

**Files in this Data Supplement:**

- **Supplementary Material**
