# Supplementary material for: Zebrafish yolk lipid processing: a tractable tool for the study of vertebrate lipid transport and metabolism
Source: Dis Model Mech. 2014 May 8;7(7):915–27. doi: 10.1242/dmm.015800 (PMC4073280; doi:10.1242/dmm.015800)
Supplement: Supplementary Material [file supp_7.7.915_DMM015800.pdf]

Resource Impact

Background:

Abnormal serum lipid levels (dyslipidemias) are important risk factors for cardiovascular and other atherosclerotic diseases. Drugs that normalize lipid abnormalities are recognized as essential in the treatment of these diseases. However, many lipid disorders are complex, resistant to simple treatment strategies. While diet certainly contributes to the development of dyslipidemias, there are also important genetic factors at play. Since lipid disorders are under complex genetic and physiological regulation, a thorough understanding of these mechanisms is essential for developing improved therapies. Thus, researchers study animal models that share genetic and physiological traits with human but can also be manipulated genetically. The mouse model system is commonly used to understand lipoprotein metabolism, yet mice lack an enzyme critical for cholesterol transfer between lipoprotein classes (cholesterol ester transfer protein, CETP). Interestingly, the zebrafish model is emerging as a notable system for studies in lipid metabolism. Zebrafish are easy to manipulate genetically and share the fundamentals of lipid and lipoprotein biology with humans, including a CETP homolog.

Results:

In this study, the authors describe the value of the yolk metabolizing stages of the zebrafish as a model for studying lipid and lipoprotein metabolism. The authors inject fatty acids tracers into the yolk of living zebrafish embryos and larvae. The tracers enable researchers to follow the fate of fatty acids as they are metabolized, packaged into lipoproteins, excreted into the circulation and absorbed by the tissues of the body. Using a genetic model for abetalipoproteinemia, the authors show that secretion of labeled fatty

acids into the circulation is dependent on lipoprotein production. Additionally, they examine the metabolic fate of exogenously delivered fatty acids by assaying their incorporation into complex lipids. Finally, they demonstrate that using this technique, they can detect lipid/lipoprotein abnormalities caused by a genetic mutation (microsomal triglyceride transfer protein) and an inhibitor of a lipid-metabolizing enzyme (acyl-CoA:cholesterol acyltransferase).

Implications and future directions:

The authors' results suggest that zebrafish yolk lipid transport and metabolism is a tractable model to study lipid and lipoprotein biology, regulation, and metabolism. Zebrafish at these early stages are relatively simple, lacking many features that complicate the interpretation of metabolic studies. This readout of physiology can be readily combined with pharmacological and/or genetic tools to study the fundamentals of lipid metabolism. Moreover, targeting genes that result in early lethality or defects in the development of the liver and/or intestine would be possible using these techniques.

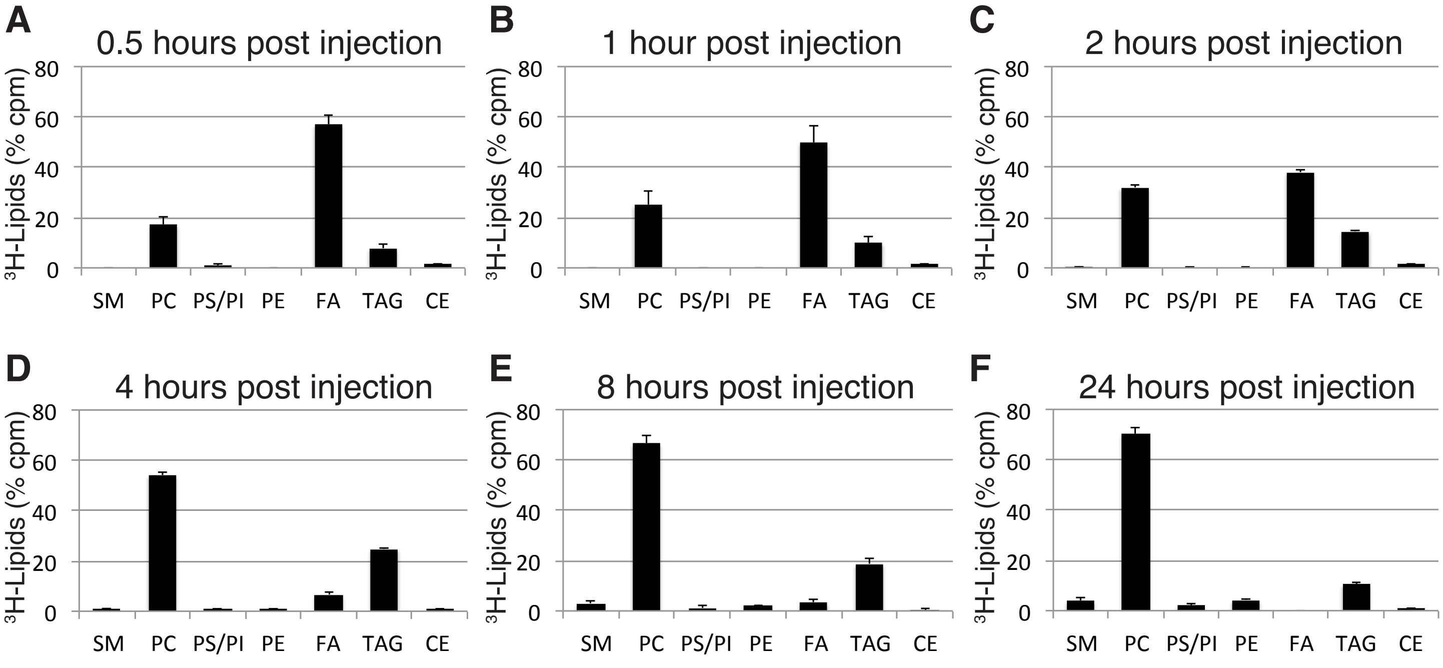

Supplemental Figure 1: Time course of palmitate absorption and incorporation.

Incorporation and utilization of radiolabeled neutral lipids and phospholipids at various time points after injection of 3H-palmitate into the yolk of 3-dpf larvae. Data is represented as mean ± SE of total radioactive counts.

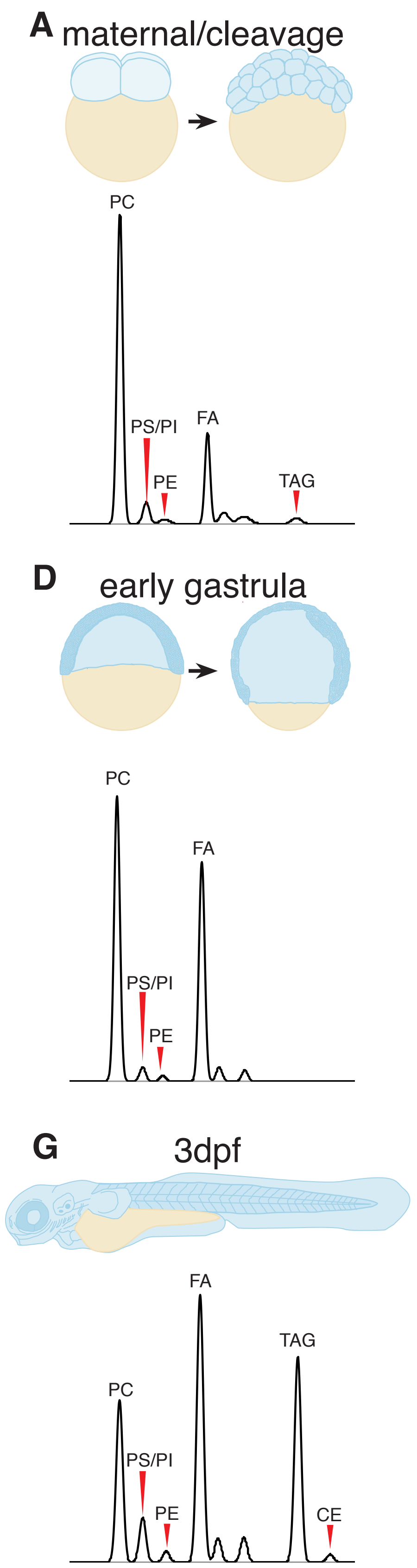

Supplemental Figure 2: Developmental analysis of fatty acid metabolism.

Diagrams of the developmental stages analyzed and the corresponding representative chromatographs showing radioactive peaks of 3H-oleic acid metabolites.

	14:0	14:1n5	15:0	pm16:0	16:0	*trans*16:1n7	16:1n7	pm18:0	pm18:1n9	pm18:1n7
Cholesterol Ester	33 ± 25	0 ± 0	17 ± 15	1 ± 1	106 ± 32	3 ± 3	10 ± 4	0 ± 0	0 ± 0	0 ± 0
Cardiolipin	21 ± 9	1 ± 1	12 ± 4	6 ± 5	211 ± 78	4 ± 4	21 ± 2	5 ± 3	3 ± 2	1 ± 0
Diacylglycerol	10 ± 6	1 ± 1	5 ± 3	2 ± 1	84 ± 38	6 ± 5	4 ± 2	0 ± 0	0 ± 0	0 ± 0
Free fatty acid	22 ± 12	3 ± 3	10 ± 6	0 ± 0	167 ± 82	13 ± 9	12 ± 6	0 ± 0	0 ± 0	0 ± 0
Lysophosphatidylcholine	8 ± 4	0 ± 0	3 ± 2	1 ± 0	75 ± 35	8 ± 8	1 ± 0	2 ± 2	0 ± 0	0 ± 0
Phosphatidylcholine	25 ± 3	0 ± 0	32 ± 0	9 ± 5	2109 ± 58	17 ± 17	64 ± 17	2 ± 1	0 ± 0	0 ± 0
Phosphatidylethanolamine	12 ± 6	2 ± 2	7 ± 3	97 ± 31	331 ± 89	7 ± 7	8 ± 2	40 ± 15	7 ± 1	4 ± 1
Phosphatidylserine	17 ± 12	1 ± 0	9 ± 6	7 ± 4	168 ± 70	16 ± 16	2 ± 1	5 ± 3	2 ± 2	3 ± 3
Sphingomyelin	33 ± 13	7 ± 6	26 ± 9	5 ± 5	428 ± 335	10 ± 10	15 ± 6	0 ± 0	0 ± 0	0 ± 0
Triacylglycerol	25 ± 3	3 ± 1	17 ± 6	18 ± 18	304 ± 116	8 ± 8	39 ± 18	5 ± 5	2 ± 0	0 ± 0

	18:0	18:1n9	18:1n7	*trans*18:2n6	18:2n6	18:3n6	18:3n3	18:4n3	20:0	20:1n9
Cholesterol Ester	57 ± 11	72 ± 12	7 ± 3	1 ± 1	36 ± 14	2 ± 2	5 ± 3	1 ± 1	3 ± 0	4 ± 3
Cardiolipin	184 ± 64	100 ± 21	72 ± 9	0 ± 0	71 ± 39	5 ± 5	10 ± 3	21 ± 20	4 ± 2	11 ± 4
Diacylglycerol	58 ± 32	32 ± 13	6 ± 2	0 ± 0	33 ± 22	5 ± 5	1 ± 1	4 ± 3	4 ± 3	1 ± 1
Free fatty acid	100 ± 37	86 ± 50	11 ± 5	0 ± 0	46 ± 39	1 ± 1	3 ± 2	1 ± 1	4 ± 2	3 ± 2
Lysophosphatidylcholine	37 ± 17	28 ± 13	4 ± 2	0 ± 0	6 ± 3	0 ± 0	1 ± 1	1 ± 0	2 ± 1	2 ± 1
Phosphatidylcholine	376 ± 33	923 ± 43	96 ± 3	0 ± 0	132 ± 56	5 ± 2	17 ± 3	4 ± 1	17 ± 2	13 ± 1
Phosphatidylethanolamine	693 ± 205	160 ± 56	39 ± 9	0 ± 0	21 ± 8	6 ± 5	9 ± 7	4 ± 3	14 ± 4	7 ± 2
Phosphatidylserine	527 ± 202	183 ± 83	22 ± 13	0 ± 0	19 ± 9	0 ± 0	12 ± 11	0 ± 0	16 ± 13	6 ± 2
Sphingomyelin	115 ± 88	99 ± 74	27 ± 25	19 ± 19	12 ± 8	8 ± 7	2 ± 2	3 ± 2	11 ± 6	4 ± 2
Triacylglycerol	118 ± 45	158 ± 50	37 ± 17	0 ± 0	40 ± 9	3 ± 1	9 ± 5	1 ± 1	6 ± 0	6 ± 1

	20:3n9	20:2n6	20:3n6	20:4n6	20:3n3	20:4n3	20:5n3	22:0	22:1n9	22:2n6
Cholesterol Ester	0 ± 0	3 ± 2	7 ± 4	21 ± 8	1 ± 1	3 ± 1	19 ± 5	2 ± 1	4 ± 1	0 ± 0
Cardiolipin	5 ± 2	12 ± 2	10 ± 5	39 ± 15	4 ± 4	14 ± 10	22 ± 2	4 ± 2	31 ± 10	18 ± 15
Diacylglycerol	6 ± 6	15 ± 15	5 ± 5	8 ± 5	0 ± 0	2 ± 2	9 ± 2	2 ± 1	7 ± 6	9 ± 9
Free fatty acid	13 ± 13	0 ± 0	1 ± 1	15 ± 8	0 ± 0	11 ± 11	16 ± 11	5 ± 2	7 ± 5	0 ± 0
Lysophosphatidylcholine	2 ± 2	3 ± 1	0 ± 0	3 ± 1	0 ± 0	7 ± 7	4 ± 2	1 ± 1	1 ± 1	2 ± 2
Phosphatidylcholine	2 ± 1	12 ± 1	31 ± 12	148 ± 14	2 ± 2	9 ± 2	257 ± 43	3 ± 1	6 ± 1	1 ± 0
Phosphatidylethanolamine	14 ± 11	3 ± 1	10 ± 3	209 ± 67	1 ± 1	3 ± 1	127 ± 18	2 ± 1	7 ± 2	0 ± 0
Phosphatidylserine	1 ± 1	3 ± 2	6 ± 3	35 ± 20	3 ± 3	5 ± 3	26 ± 8	3 ± 2	7 ± 5	17 ± 16
Sphingomyelin	9 ± 9	6 ± 1	2 ± 2	13 ± 8	4 ± 2	3 ± 1	26 ± 15	15 ± 6	7 ± 2	0 ± 0
Triacylglycerol	1 ± 0	5 ± 1	6 ± 1	28 ± 14	0 ± 0	6 ± 3	82 ± 54	3 ± 1	3 ± 2	1 ± 1

	22:4n6	22:5n6	22:5n3	22:6n3	24:0	24:1n9	24:6n3
Cholesterol Ester	1 ± 1	1 ± 1	6 ± 2	68 ± 15	2 ± 2	2 ± 2	0 ± 0
Cardiolipin	1 ± 1	2 ± 2	16 ± 2	223 ± 38	4 ± 2	4 ± 3	0 ± 0
Diacylglycerol	0 ± 0	0 ± 0	1 ± 1	25 ± 5	1 ± 1	1 ± 0	0 ± 0
Free fatty acid	2 ± 2	0 ± 0	4 ± 3	42 ± 25	2 ± 1	3 ± 1	0 ± 0
Lysophosphatidylcholine	1 ± 1	0 ± 0	2 ± 1	20 ± 7	1 ± 0	0 ± 0	0 ± 0
Phosphatidylcholine	12 ± 6	12 ± 12	72 ± 16	1026 ± 60	7 ± 0	4 ± 3	0 ± 0
Phosphatidylethanolamine	12 ± 8	5 ± 5	56 ± 14	1037 ± 214	12 ± 1	0 ± 0	3 ± 3
Phosphatidylserine	45 ± 35	4 ± 4	65 ± 10	670 ± 238	0 ± 0	0 ± 0	4 ± 4
Sphingomyelin	1 ± 0	0 ± 0	13 ± 10	71 ± 56	11 ± 4	9 ± 3	1 ± 1
Triacylglycerol	7 ± 2	3 ± 3	24 ± 15	157 ± 111	7 ± 4	1 ± 1	0 ± 0

Supplemental Table 1: Larval acyl chain profiles of major lipid classes. Lipidomic data are expressed as pmol/larvae (6 dpf stage; 150 larvae/sample, n = 3). Data are represented as mean ± SE.

	% total lipids	% palmitate 16:0	% oleate 18:1n9	% lignocerate 24:0
Cholesterol Ester	6.0 ± 1.3	2.7 ± 0.8	3.9 ± 0.7	5.2 ± 4.5
Cardiolipin	3.5 ± 1.0	5.3 ± 2.0	5.4 ± 1.1	7.6 ± 5.0
Diacylglycerol	2.1 ± 1.0	2.1 ± 1.0	1.8 ± 0.7	2.0 ± 1.5
Free fatty acid	7.3 ± 3.4	4.2 ± 2.1	4.7 ± 2.7	4.7 ± 2.8
Lysophosphatidylcholine	2.7 ± 1.2	1.9 ± 0.9	1.5 ± 0.7	1.6 ± 0.8
Phosphatidylcholine	32.9 ± 0.3	53.0 ± 1.5	50.1 ± 2.3	15.5 ± 1.0
Phosphatidylethanolamine	17.9 ± 4.2	8.3 ± 2.2	8.7 ± 3.0	25.3 ± 2.9
Phosphatidylserine	11.5 ± 4.6	4.2 ± 1.7	9.9 ± 4.5	0.9 ± 0.7
Sphingomyelin	11.4 ± 8.5	10.7 ± 8.4	5.4 ± 4.0	23.0 ± 8.3
Triacylglycerol	4.6 ± 1.7	7.6 ± 2.9	8.6 ± 2.7	14.2 ± 9.3

Supplemental Table 2: Larval lipid composition as a mole percentage of total lipid class and with specific acyl chains. Data is represented as mean percent ± SE (6 dpf stage; 150 larvae/sample, n = 3).
